# The BpMYB4 Transcription Factor From *Betula platyphylla* Contributes Toward Abiotic Stress Resistance and Secondary Cell Wall Biosynthesis

**DOI:** 10.3389/fpls.2020.606062

**Published:** 2021-01-18

**Authors:** Ying Yu, Huizi Liu, Nan Zhang, Caiqiu Gao, Liwang Qi, Chao Wang

**Affiliations:** ^1^State Key Laboratory of Tree Genetics and Breeding, School of Forestry, Northeast Forestry University, Harbin, China; ^2^Chinese Academy of Forestry, Beijing, China

**Keywords:** BpMYB4 transcription factors, abiotic stress, secondary cell wall, functional analysis 2, *Betula platyphylla* Suk

## Abstract

The MYB (v-myb avian myeloblastosis viral oncogene homolog) family is one of the largest transcription factor families in plants, and is widely involved in the regulation of plant metabolism. In this study, we show that a MYB4 transcription factor, BpMYB4, identified from birch (*Betula platyphylla* Suk.) and homologous to EgMYB1 from *Eucalyptus robusta* Smith and ZmMYB31 from *Zea mays* L. is involved in secondary cell wall synthesis. The expression level of *BpMYB4* was higher in flowers relative to other tissues, and was induced by artificial bending and gravitational stimuli in developing xylem tissues. The expression of this gene was not enriched in the developing xylem during the active season, and showed higher transcript levels in xylem tissues around sprouting and near the dormant period. *BpMYB4* also was induced express by abiotic stress. Functional analysis indicated that expression of *BpMYB4* in transgenic Arabidopsis (*Arabidopsis thaliana*) plants could promote the growth of stems, and result in increased number of inflorescence stems and shoots. Anatomical observation of stem sections showed lower lignin deposition, and a chemical contents test also demonstrated increased cellulose and decreased lignin content in the transgenic plants. In addition, treatment with 100 mM NaCl and 200 mM mannitol resulted in the germination rate of the over-expressed lines being higher than that of the wild-type seeds. The proline content in transgenic plants was higher than that in WT, but MDA content was lower than that in WT. Further investigation in birch using transient transformation techniques indicated that overexpression of *BpMYB4* could scavenge hydrogen peroxide and O_2_^.–^ and reduce cell damage, compared with the wild-type plants. Therefore, we believe that BpMYB4 promotes stem development and cellulose biosynthesis as an inhibitor of lignin biosynthesis, and has a function in abiotic stress resistance.

## Introduction

As a ubiquitous transcription factor in plants and animals, MYB proteins are widely involved in the regulation of developmental and metabolic changes in plants. The first *MYB* gene in plants, *ZmMYBC1*, was isolated from maize (Paz-ares et al., 1987). Subsequently, a large number of *MYB* functional genes were isolated and identified from various plants (Dubos et al., 2010). There are at least 196 *MYB* genes in Arabidopsis (*Arabidopsis thaliana*), at least 197 in Populus (*Populus trichocarpa*), and 124 in grapevine (*Vitis vinifera* L.) (Wilkins et al., 2009).

NAC-MYB-based transcriptional regulation of secondary cell wall biosynthesis in land plants is widely known. Plant secondary growth processes are affected by both MYB transcriptional activators and MYB transcriptional repressors, most of which belong to the fourth sub-group of the MYB family (Chen et al., 2002; Vannini et al., 2004). As transcriptional activators, AtMYB46 has been well-studied. Two highly homologous Arabidopsis genes, *AtMYB46* and *AtMYB83*, can be activated by *SND1* (SECONDARY WALL-ASSOCIATED NAC DOMAIN PROTEIN 1) and its homologous proteins, and its overexpression can activate the biosynthesis of lignin, cellulose and hemicellulose (Zhong et al., 2007; Mccarthy et al., 2009). *AtMYB26* regulates the synthesis of secondary cell walls through *NST1* (NAC SECONDARY WALL THICKENING PROMOTING FACTOR 1) and *NST2*, and its overexpression can lead to ectopic deposition of secondary cell walls (Mitsuda et al., 2005; Yang et al., 2007). *PtMYB4* from pine (*Pinus taeda*) and *EgMYB2* from eucalyptus (*Eucalyptus robusta Smith*) belong to the same phylogenetic group as *AtMYB46*. *PtMYB1* and *PtMYB4* can bind with AC elements and are expressed in developing wood, causing secondary wall thickening and lignin biosynthesis (Patzlaff et al., 2003b). Overexpression of *PtMYB4* in tobacco (*Nicotiana tabacum* L.) plants can induce the expression of some lignin biosynthesis genes and cause abnormal deposition of lignin and secondary wall thickening (Patzlaff et al., 2003a). *PtrMYB003* and *PtrMYB020* in Populus are homologous with Arabidopsis *AtMYB46* and are specifically expressed in secondary xylem; when overexpressed in Arabidopsis they activate the biosynthesis of cellulose, hemicellulose, and lignin (Mccarthy et al., 2010). Overexpression of *AtMYB58* and *AtMYB63* promoted the expression of lignin biosynthesis genes, resulting in ectopic deposition of lignin; in contrast, inhibiting their expression reduced the lignin content, resulting in secondary cell wall developmental defects (Zhong et al., 2008; Zhou et al., 2009). In addition, the MYB transcription factor regulates the synthesis of secondary walls by participating in other phenylpropanoid pathways. For example, AtMYB75 mainly regulates the biosynthesis of anthocyanins, but overexpression of this protein also leads to a slight increase in lignin accumulation, indicating that the protein is also involved in the regulation of lignin biosynthesis (Borevitz et al., 2000).

MYBs can also act as transcriptional inhibitors of lignin biosynthesis. For example, transfer of the Antirrhinum (*Antirrhinum majus* L.) *AmMYB308* gene into tobacco resulted in a significant decrease in the expression of C4H (cinnamate-4-hydroxylase), 4CL (4-coumarate:coenzyme A ligase) and CAD (cinnamyl alcohol dehydrogenase) genes, there by effectively inhibiting the biosynthesis of lignin. Transfer of *AmMYB300* into tobacco resulted in the inhibition of expression of 4CL (Tamagnone et al., 1998). In Arabidopsis, overexpression of the maize (*Zea mays* L.) transcriptional repressors ZmMYB31 and ZmMYB42 resulted in the inhibition of expression of *COMT* (caffeic acid 3-O-methyltransferase), thereby reducing lignin content (Fornalé et al., 2006). *EgMYB1* from eucalyptus is an inhibitor of lignin biosynthesis (Legay et al., 2007). Overexpression of *PvMYB4* in switchgrass (*Panicum virgatum*) inhibits lignin biosynthesis (Shen et al., 2011). Overexpression of *AtMYB32* causes anther distortion and affects the formation of anther cell walls (Preston et al., 2004). MYB189 negatively regulates secondary cell wall biosynthesis in Populus (Jiao et al., 2019). Given that there is a little information on the function of negative regulators in woody plants, the screening and identification of MYB transcription factors with transcriptional activation or transcriptional inhibition has broad research significance.

Following a stress stimulus, the expression of some transcription factors increases, resulting in signal amplification. Thus, the expression of downstream related genes are also regulated, so that overall stress resistance is improved to some extent. According to existing reports, MYB transcription factors are involved in a variety of plant stress responses and are key in regulating the cell’s response to stress. Examples include a high temperature resistance gene *MYB68* (Feng et al., 2004); genes that respond to drought and salt stress, *GmMYB177* (Liao et al., 2008); genes that respond to drought and cold stress, *OsMYB4*; genes that respond to drought, salt and radiation stress, *OsMYB4* (Pasquali et al., 2008); genes that respond to cold, salt and drought stress, *AtMYB2* (Hoeren et al., 1998); and *OsMYB2* (Yang et al., 2012), to name a few.

Secondary growth and stress resistance are the important physiological processes for tree radial growth and wood formation. The properties of wood are determined by the composition and characteristics of xylem secondary cell wall. For pulping industry the lignin content in secondary cell wall is an important factor affecting pulping yield. The birch (*Betula platyphylla* Suk.) is one of the main pulpwood species. Therefore, it is of great significance to study the lignin biosynthesis and stress resistance for the genetic improvement of forest trees. In this study, a MYB transcription factor homologous to the genes inhibiting lignin synthesis was identified. The expression pattern of this gene in tissues, under artificial bending or abiotic stress treatment were analyzed. The functions related to secondary cell wall biosynthesis were identified by over-expressing *BpMYB4* in *Arabidopsis thaliana*. Besides, the function of this gene in abiotic stress tolerance was investigated through transient transformation in birch. The expression patterns of genes involved in abiotic stress response and cell wall biosynthesis in transgenic plants were analyzed. The results can help further understand the molecular mechanism of secondary growth processes and abiotic stress responses regulated by MYB transcription factors in woody plants.

## Materials and Methods

### Plant Materials and Treatment

Under aseptic conditions, the epidermal cells of onion were torn and plated in 1/2 MS solid medium for sub-cellular localization studies (Wang et al., 2019).

The vernalized birch (*B. platyphylla*) seeds were washed with running water for 3 days and planted on the soil surface; the soil contained a mixture of perlite: vermiculite: soil (1: 1: 4). The plants were grown in a greenhouse under controlled conditions (16 h/8 h light/dark, 25°C, 70–75% relative humidity). Two-month-old seedlings were used to test the express levels between leaves, stems and roots. Developing xylem of 2-years-old birch seedlings were subjected to artificial bending experiments and temporal expression analysis (Wang et al., 2014). The birch seedlings were irrigated with 200 mM NaCl or 300 mM Mannitol solutions for 12 h, 24 h, and 48 h, and the birch irrigated with water was used as a control group for expression analysis under abiotic stress. Three biological replicates were performed.

Wild-type (WT) *Arabidopsis thaliana* (ecotype Col-0) seeds were surface-sterilized with 50% sodium hypochlorite for 6 min, washed six times with sterile distilled water, the seeds plated on solid 1/2 MS medium and incubated at 22°C under light. After growing the cotyledons, the seedlings were transferred to a mixture of perlite: vermiculite: soil (1: 1: 4) and grown in a greenhouse under controlled conditions (16 h/8 h light/dark, 25°C, 70–75% relative humidity). The plants were thoroughly watered every 5 days (Wen and Chang, 2002).

### Gene Identification and Bioinformatics Analysis

A cDNA sequence (GenBank accession number KA257119.1) annotated as a MYB transcription factor was screened from birch tension wood transcriptome (Wang et al., 2014). The open reading frame (ORF) was determined using the NCBI blastX procedures^1^. The molecular weight and isoelectric point of the protein were predicted using Expasy^2^. The similarities in amino acid sequences were checked using BioEdit for multiple sequence alignments, and finally the MEGA version 4 software (Kubo et al., 1998) was used for phylogenetic tree analysis.

### Sub-Cellular Localization

Total RNA of birch was extracted using CTAB method (Chang et al., 1993) and treated with DNase I to remove DNA contamination. Total RNA was reverse transcribed into cDNA using a PrimeScriptTMRT reagent Kit (Takara Bio Inc., Kusatsu, Shiga, Japan). *BpMYB4* CDS without a stop codon was fused to the N-terminus of green fluorescent protein (GFP) into the pROKII vector and driven by a CaMV 35S promoter. Vector construct primers are shown in Supplementary Table 1. The microcarriers (tungsten powder) embedded with the 35S: GFP plasmid and the 35S: gene-GFP plasmid were transformed into onion epidermal cells with high pressure helium gas using a PDS-1000 benchtop gene gun (Bio-Rad laboratories, Inc., Hercules, CA, United States). After 2 days of culture under dark conditions, the expression pattern was observed and captured using a LSM700 laser confocal microscope (Zeiss, Jena, Germany).

### Real-Time PCR

Total RNA of plant was extracted by CTAB method for semi-quantitative RT-PCR and real-time RT-PCR analysis. Real-time RT-PCR was performed with a TransStart Top Green qPCR SuperMix kit (TransGen Biotech, Beijing, China) using the primer sequences listed in Supplementary Table 2. The amplification procedure was conducted using the following parameters: 94°C for 30 s; 45 cycles at 94°C for 12 s, 58°C for 30 s and 72°C for 45 s; and 79°C for 1 s for plate reading. The semi-quantitative PCR enzyme by rTaq (TaKaLa), which amplification procedure was conducted using the following parameters: 94°C for 2 min; 28 cycles at 94°C for 30 s, 58°C for 30 s and 72°C for 45 s. Three independent experiments were performed. The tubulin (GenBank accession number: FG067376) and ubiquitin (GenBank accession number: FG065618) genes were used as the internal controls. The relative expression level of each gene was calculated using the delta–delta CT method (Livak and Schmittgen, 2001).

### Construction of Overexpression and Knock-Down Expression Vectors

The CDS of genes was inserted into the pROKII vector to construct the overexpression vector (OE) driven by a CaMV 35S promoter. A short sequence inverted repeat of 200 bp was inserted in the RNAi vector pFGC5941 to generate the RNAi-silencing knock down vector (SE). Vector construct primers are shown in Supplementary Table 1. The recombinant plasmids of the overexpression vector or the RNAi-silencing vector were transformed into *Agrobacterium tumefaciens* strain EHA105 using the freeze-thaw method (Zhang et al., 2018).

### Transformation of Arabidopsis

For transformation, WT Arabidopsis was used at flowering time with the ripe pods removed. Transformation of 35S: MYB4-GFP into *A. thaliana* was performed using the Arabidopsis flower dipping method (Clough and Bent, 1998). The obtained seeds were screened for resistance with 1/2 MS solid medium containing 50 mg/L kanamycin, until T3 generation seeds were obtained. The semi-quantitative PCR was performed for expression identification of *BpMYB4* in transgenetic *Arabidopsis*. Primers are shown in Supplementary Table 2.

### Histochemical Analysis

Transgenic *A. thaliana* growing for about 50 days was selected as the experimental group, and WT *A. thaliana* in the same growth cycle under the same environment was used as the control group. The mature stems of *A. thaliana* in the control group and the experimental group were sectioned by hand, and histochemically stained with a 1% phloroglucinol solution. The sections were observed with an optical microscope (Olympus BX53, Japan).

### Chemical Analysis of Secondary Cell Wall

Lignin and cellulose content were determined by the Soxhlet extraction method (Thurbide and Hughes, 2000). The transgenic and WT Arabidopsis plants were harvested at about 50 days growth stage, dried in an oven at 105°C, pulverized, and bake to a constant weight at 55°C. A sample weight of exactly 0.5 g (weight to 0.0001 g) was used to determine the content of lignin in transgenic Arabidopsis, and a sample weight of exactly 1.0 g (weight to 0.0001 g) was used to determine the content of cellulose. Three technical replicas were made. All samples were wrapped with filter paper and tied with cotton thread.

### Seed Germination Rate Under Abiotic Stress

Seeds of WT *A. thaliana* and from the overexpression lines of transgenic *A. thaliana* T3 generation (OE-7, OE-9, OE-25) were washed with sodium hypochlorite solution and sequentially sown on 1/2 MS, 1/2 MS + 100 mM NaCl, and 1/2 MS + 200 mM mannitol (Ji et al., 2013). The seeds were germinated in an artificial climate culture chamber for 10–15 days, and the germination rate was counted.

### MDA and PRO Content Determination

Using PRO kit (Nanjing Jiancheng, China) to determine the content of L-proline (Pro) in plant materials under abiotic stress. Malondialdehyde (MDA) determination of plants under treatment was conducted by thiobarbituric acid method (Wang et al., 2010b). Three biological replicates were performed.

### Transient Transformation in Birch and Treatment of Plants

*Agrobacterium tumefaciens* with pROKII, pFGC5941 empty vector, and the recombinant vectors were cultured to OD 600 value of 0.5, and the cells were collected and resuspended in 5% sucrose, 1.5 mg/L KT (6-Furfurylaminopurine), 0.5 mg/L NAA (1-naphthylacetic acid), 100 μM AS (acetosyringone) and 0.02% Tween 20 in 50 mL of 1/2 MS liquid medium (pH 5.6). Two month-old birch seedlings were soaked in the medium containing resuspended bacterial cells under 25°C, with constant shaking at 100 rpm for 2 h; the cultured birch seedlings were then washed 1–2 times with sterile deionized water, and the water on the birch seedlings was dried with sterile absorbent paper. The infected birch seedlings were transplanted into artificial nutrient soil (The ratio of soil, perlite and vermiculite was 5:3:2) and moisturized by mulching. The gene expression was detected by RT-PCR after culture at 25°C and light (approximately 150 μmol m^–2^ s^–1^) for 3 days (Zhang et al., 2018).

The transiently transformed birch seedlings were replanted into the artificial nutrient soil in the greenhouse. After 3 days of culture, the transiently transformed birch and the untreated WT plants were treated with 100 mM NaCl and 200 mM mannitol for 24 h, respectively (Wang et al., 2019). Total RNA was extracted from birch seedlings under stress and transcribed into cDNA.

### DAB, NBT, and Evan’s Blue Staining

DAB (3-3’-diaminobenzidine), NBT (nitro blue tetrazolium) and Evan’s Blue staining were performed on birch leaves after stress treatment (Zhang et al., 2018). The leaves were extracted and stained with DAB, NBT and Evan’s Blue stains. At 37°C, tissues were stained with DAB overnight, stained with NBT for 4 h, and stained with Evan’s Blue for 8 h. After staining, the stain was removed and decolorized. The solution (95% ethanol + 5% glycerol) was dehydrated in a boiling water bath.

## Results

### BpMYB4 Is Homologous to Lignin Biosynthetic Inhibitory Factor

An mRNA sequence, KA257119.1, 4258 bp in length, from reaction wood of *B. platyphylla* was found in the Transcriptome Shotgun Assembly database (Wang et al., 2014). According to BLASTX and ORF analysis, a MYB transcription factor was identified with an open reading region 654 bp in length, encoding a total of 217 amino acids. The molecular weight of this MYB protein is predicted to be approximately 53.661 kDa.

**FIGURE 1:**
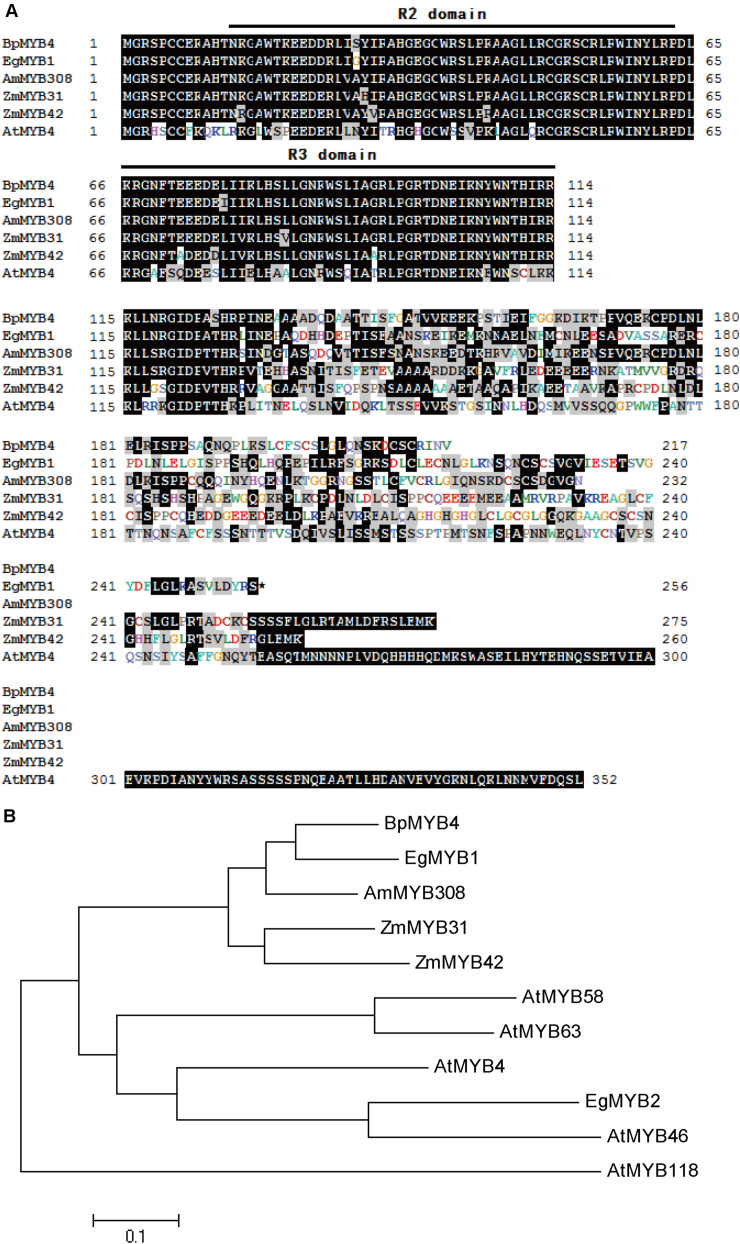
Sequence analysis of BpMYB4. **(A)** ClustalW alignment of EgMYB1 and homologous proteins using BioEdit. Black and gray shadings represent identical and similar amino acids, respectively. The conserved regions R2 and R3 MYB DNA binding domains are indicated at the top. **(B)** Neighbor joining tree of BpMYB4 and homologous proteins (Bootstrap = 1000). Full-length amino acid sequences were used to construct the tree with the Mega 5.0 software. GenBank accession numbers: EgMYB1 (CAE09058), EgMYB2 (AJ576023), ZmMYB31 (CAJ42202), ZmMYB42 (CAJ42204), AmMYB308 (P81393), AtMYB4 (AF062860), AtMYB46 (NM121290), AtMYB58 (AF062893), AtMYB63 (AF062898), AtMYB118 (AF334817).

Multi-sequence alignment analysis demonstrated (Wang et al., 2018) that BpMYB4 has a typical R2R3 MYB TF signature. BpMYB4 protein is highly similar to AmMYB308 (P81393) (Tamagnone et al., 1998), ZmMYB42 (CAJ42204) (Fornalé et al., 2006), ZmMYB31 (CAJ42202) (Fornalé et al., 2010), and EgMYB1 (CAE09058) (Legay et al., 2007) across conserved domains (Figure 1A), with similarity reaching up to 80% using the full amino acid sequence and 95% in conserved domains. A phylogenetic tree (Figure 1B) analysis showed that BpMYB4 was more similar to EgMYB1, which is a inhibitory of lignin biosynthesis, compared with Antirrhinum AmMYB308 and maize ZmMYB31 and ZmMYB42.

### Sub-Cellular Localization of BpMYB4

**FIGURE 2:**
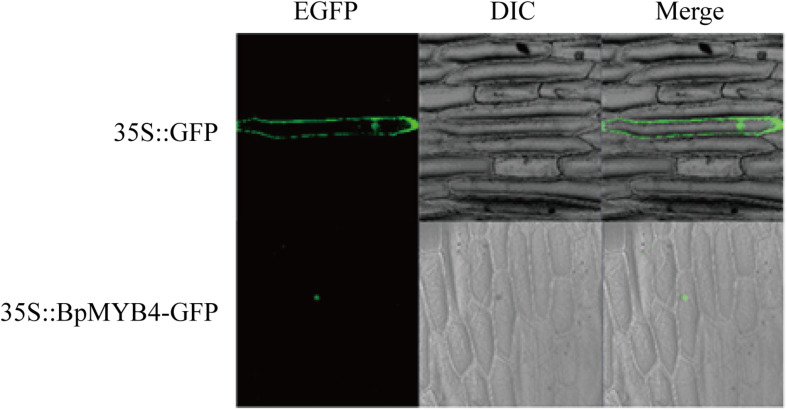
Sub-cellular localization of BpMYB4 protein in onion epidermal cells. Onion epidermal cells were transiently transformed with constructs containing vector 35S: BpMYB4-GFP through particle bombardment method. Sub-cellular localization of 35S: BpMYB4-GFP fusion proteins were viewed using fluorescent confocal microscopy.

Recombinant plasmid 35S:MYB4-GFP was transfected into onion epidermal cells using the gene gun transformation technique, and the 35S: GFP plasmid was used as a positive control. Observations using a laser confocal microscope showed a green fluorescent signal in the nucleus of the onion epidermis, indicating that BpMYB4 is a nuclear protein (Figure 2).

### Expression Analysis of BpMYB4 Gene

**FIGURE 3:**
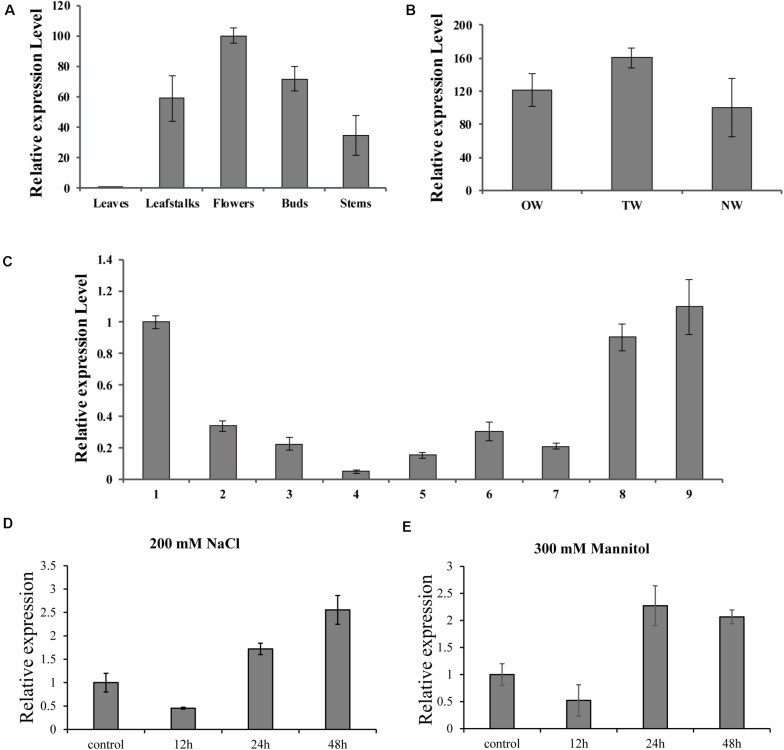
Real-time RT-PCR expression analysis of BpMYB4. **(A)** Real-time RT-PCR analysis of BpMYB4 expression in different tissues. **(B)** Real-time RT-PCR expression analysis of BpMYB4 in OW (opposite wood), TW (tension wood) and NW (normal wood). **(C)** Real-time RT-PCR analysis of BpMYB4 expression in birch xylem at different growth stages (Numbers 1–9 indicate different months). **(D)** Real-time RT-PCR analysis of BpMYB4 expression in birch under different time stresses of 200 mM NaCl. **(E)** Real-time RT-PCR analysis of BpMYB4 expression in birch under different time stresses of 300 mM Mannitol. Error bars represent standard error for three replicates.

To further investigate the biological role of *BpMYB4* in secondary growth of birch, its gene expression pattern in different tissues, development stages in a growth season and during tension wood development, were analyzed using real-time quantitative PCR. The results (Figure 3A) showed that the expression level of *BpMYB4* was the highest in the inflorescence than other tissues, followed by buds, petioles, stems and leaves of birch. Its expression level was nearly 100 times higher in inflorescence than that in leaves, and 40–60 times higher in petioles and stems, compared to leaves. In a growth season the expression level of *BpMYB4* was different in different stages of xylem development in birch (Figure 3C); its expression was abundant in late April and middle September, but decreased during active stages of cambium development. When the stems of birch were subjected to artificial bending and gravity stimulation, the transcript of *BpMYB4* was increased during tension wood (xylem formed above the area of the bending) development, compared to those in opposite wood (xylem formed below the area of the bending) and normal wood (xylem of the straight tree) (Figure 3B). Temporal expression patterns of the BpMYB4 gene were carried out to test BpMYB4 response to salt stress and drought stress using qRT-PCR. The results showed the expression of BpMYB4 were induced at 24 h after stress treatment (Figures 3D,E).

### Phenotypic Analysis by Ectopic Expression of BpMYB4 in Transgenic Arabidopsis

**FIGURE 4:**
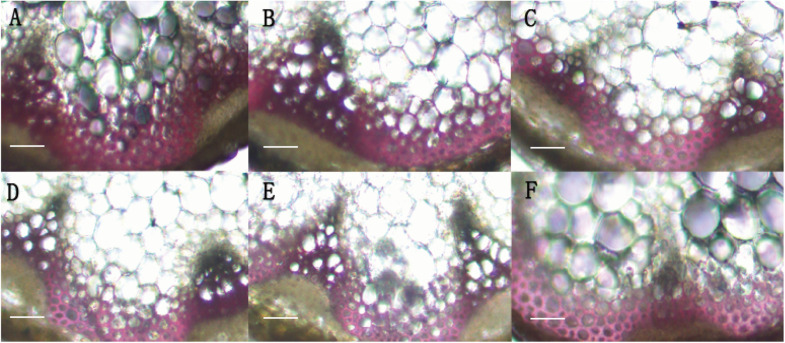
Histological observation of transgenic Arabidopsis. **(A)** Histological observation of WT Arabidopsis. **(B–F)** Histological observation of transgenic Arabidopsis stems (**B:** line 2; **C:** line 3; **D:** line 7; **E:** line 9; **F:** line 25). scale bar represent 50 μm.

In order to identify the function of *BpMYB4* in secondary growth, transgenic Arabidopsis plants overexpressing *BpMYB4* were generated. The semi-quantitative PCR was used to verify the expression of BpMYB4 gene in transgenic Arabidopsis, The results (Supplementary Figure 1) showed that *BpMYB4* gene were expressed in transgenic line 7, line 9 and line 25. The phenotypic observation showed the growth rate of the inflorescence stems of transgenic plants increased (Supplementary Figure 2B) compared with the WT. Transgenic Arabidopsis had a 10–15 day longer life cycle than WT. We further detected lignin deposition using anatomical observations (Figure 4). The stem sections stained with phloroglucinol-HCl showed decreased lignin deposition in transgenic plants with lighter red stain, compared to WT *A. thaliana*.

### Analysis of Lignin and Cellulose Content in Transgenic *A. thaliana*

To verify the inhibitory role of *BpMYB4* in lignin biosynthesis, chemical analysis was performed to detect the changes in lignin and cellulose content of transgenic Arabidopsis. Chemical composition analysis suggested that the content of lignin in transgenic lines was reduced relative to WT, with decreasement of 3.85–8.23%. However, compared with WT, the content of cellulose in transgenic plants increased significantly (*P* < 0.05), with a averange increasment of 5.29–13.15% (Table 1). These results indicated that *BpMYB4* overexpression has a negative effect on lignin biosynthesis and deposition but a positive effect on cellulose content.

**TABLE 1:** Determination of lignin and cellulose.

Number	Lignin%	Cellulose%
Line 2	12.08*	24.76*
Line 3	13.77*	25.01*
Line 7	12.81*	27.15*
Line 9	13.56*	27.81*
Line 25	13.75*	25.82*
WT	14.9	22.08

Each experiment was replicated independently for at least three times. Asterisk indicates significant difference (*p < 0.05).

### Analysis of Germination Rate in Response to Salt and Osmotic Stress

**FIGURE 5:**
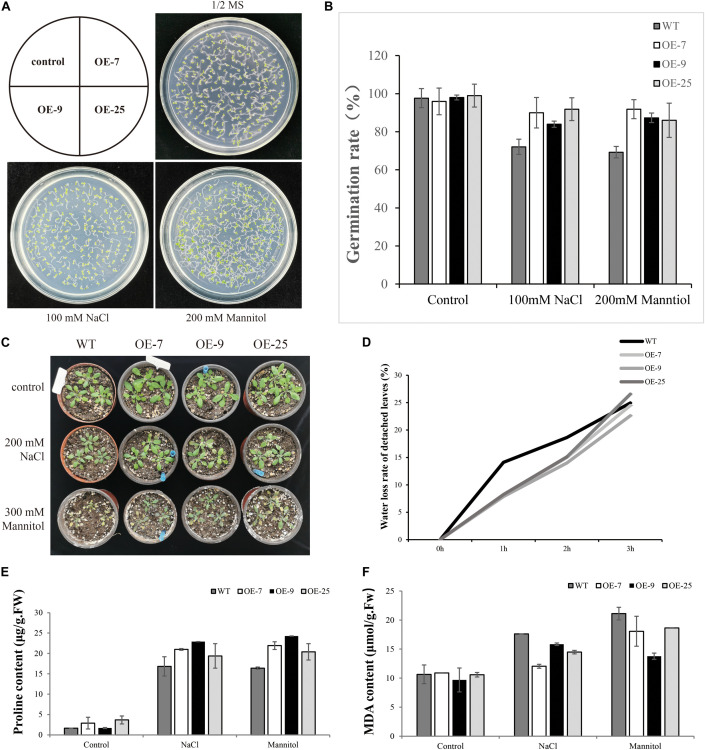
Seed germination assays in mature seeds of WT (WT) and BpMYB4-OE lines (OE-7, OE-9, and OE-25). **(A)** Germination of WT, OE-7, OE-9 and OE-25 Arabidopsis seeds on 1/2 MS media, 100 mM NaCl + 1/2 MS and 200 mM mannitol + 1/2 MS. **(B)** Quantification of greening cotyledons on plates corresponding to **(A)**. **(C)** Growth phenotype of Arabidopsis plants. **(D)** Water loss rate of detached leaves. **(E)** Determination of Pro content in Arabidopsis plants. **(F)** Determination of MDA content in Arabidopsis plants.

When the seeds of transgenic lines (OE-7, OE9, OE-25) and WT were exposed to salt or mannitol, it was found that *BpMYB4* overexpression conferred salt and osmotic stress tolerance to the seeds. There was no substantial difference in germination rates between transgenic Arabidopsis and WT lines under control conditions (Figure 5). Following 100 mM NaCl or 200 mM mannitol treatment, compared with WT plants both OE-7, OE-9 and OE-25 lines displayed higher germination rates. These results suggest that *BpMYB4* overexpression improves abiotic stress tolerance in Arabidopsis.

### MDA and PRO Content Determination

Malondialdehyde is the product of membrane lipid peroxidation in plant tissues subjected to oxidative stress under adversity, reflecting the degree of cell membrane lipid peroxidation and the strength of plant response to adversity (Ma et al., 2015). Pro plays an important role in osmotic regulation in plants. Under adversity conditions, the Pro content reflects the stress resistance of plants to a certain extent (Vendruscolo et al., 2007). We tested the content of MDA and Pro to study the resistance of *BpMYB4* transgenic plants to salt and drought stress. The WT and transgenic Arabidopsis were treated with 200 mM NaCl and 300 mM Mannitol solutions for 24 h, and the control plants were treated with water. Compared with the control group, the content of MDA in transgenic Arabidopsis was reduced, and the content of Pro was increased. The above results indicate that the *BpMYB4* gene has certain stress resistance ability.

### Generation of Transiently Transgenic Birch Plants With Knocked Down or Overexpressing BpMYB4

**FIGURE 6:**
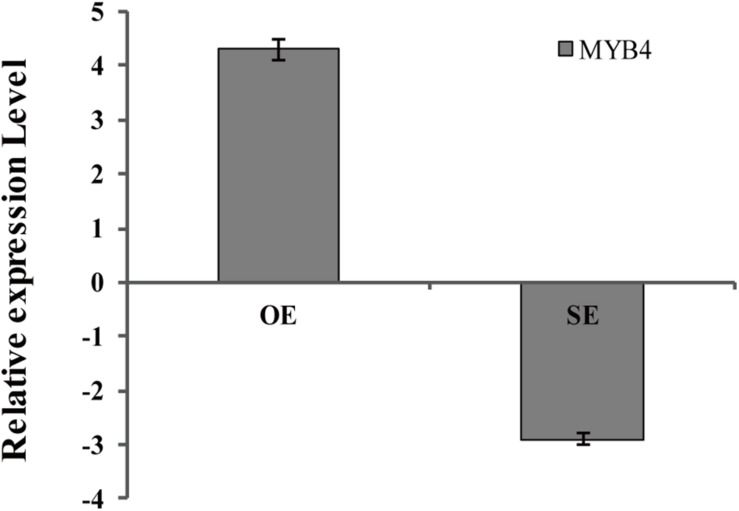
Analysis of BpMYB4 expression in birch after transient transformation. The overexpression vector 35S: BpMYB4 (OE) and the inhibitory expression vector pFGC5941-BpMYB4 (SE) were transformed into birch seedlings by infection to make the vector transiently expressed in the plant, and the expression analysis of BpMYB4 was performed by real-time quantitative PCR.

*BpMYB4* was over-expressed or knocked down in birch using an *Agrobacterium*-mediated transient expression system, and the expression levels of *BpMYB4* in transgenic plants were detected by qRT-PCR (Figure 6). The results showed that expression of *BpMYB4* was highly up-regulated in the transiently transformed plants with 35S: MYB4 vectors, which was approximately 20-fold higher than that of the control, while expression of *BpMYB4* in the transiently transformed knock-down plants (pFGC5941: MYB4) was down-regulated, with only 1/4th of the transcripts present in the control. The results showed that *BpMYB4* was overexpressed or knocked down in the transiently transformed birch seedlings. These transformed lines were used in further functional analysis.

### BpMYB4 Functions to Scavenge ROS and Maintain Cell Membrane Integrity in Transgenic Birch Subjected to Abiotic Stress

**FIGURE 7:**
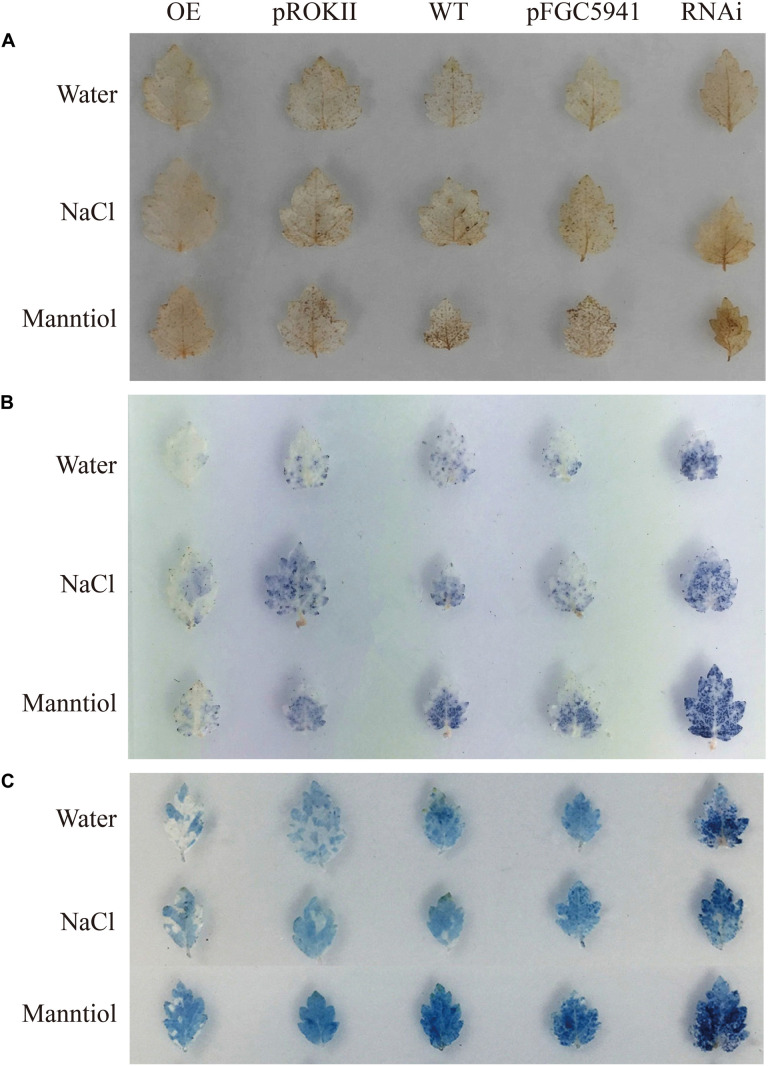
Histochemical staining analysis of transiently transformed birch. The plants were stained with DAB **(A)** and NBT **(B)** to reveal the accumulation of ${\text{O}}_{2}^{.-}$ and H_2_O_2_, respectively. **(C)** Analysis of cell death by Evan’s Blue staining.

Abiotic stress can induce the generation of reactive oxygen species (ROS), such as hydrogen peroxide (H_2_O_2_) and superoxide anion O_2_^.–^, and accumulation of ROS could damage cell membranes by oxidation of proteins, lipids and DNA (Mittler et al., 2004). To analyze the physiological mechanisms by which *BpMYB4* increases the ability to tolerate stress, birch plants were transformed transiently with 35S: BpMYB4, pROKII plasmid (control), pFGC5941: BpMYB4 or pFGC5941 plasmid (control), and WT as well as transgenic plants were irrigated with water (contro), 100 mM NaCl or 200 mM mannitol for 24 h. DAB staining in plants following abiotic stress have previously shown reduced hydrogen peroxide accumulation, leading to increased resistance (Sela et al., 2013). In the present results, DAB staining showed that under non-stress condition the over-expression (OE) plants, silencing expression (SE) plants, control plants and WT plants were not stained, with no obvious difference between them (Figure 7A). These results indicate that the content of hydrogen peroxide in these lines was similar in the controls. Under abiotic stress conditions, hydrogen peroxide accumulation in OE plants were at the lowest levels, and the hydrogen peroxide accumulation levels in the SE plants were the highest. This indicates that *BpMYB4* can reduce hydrogen peroxide accumulation in OE birch seedlings subjected to abiotic stress, and plays a role in stress tolerance of birch plants.

We performed NBT staining on the transiently transformed birch plants after abiotic stress treatment. Studies have shown that NBT staining can detect the accumulation of O_2_^.–^ in cells under stress, thereby demonstrating the resistance ability of plants (Yu et al., 2018). The results are shown in Figure 7B. Following water treatment, there was no obvious color difference between OE plants, SE plants, control group and WT, indicating that O_2_^.–^ content was basically the same. However, under NaCl or mannitol treatment, the level of O_2_^.–^ in the OE plants was the lowest, and the level of O_2_^.–^ in SE plants was the highest. This shows that the content of O_2_^.–^ in the OE plants is smaller than that in WT and control group. Our results indicate that *BpMYB4* can reduce the accumulation of O_2_^.–^ in birch trees under abiotic stress, further suggesting that *BpMYB4* plays a role in the stress resistance of birch trees.

Studies have shown that Evan’s Blue staining can detect cell death and demonstrate the degree of stress resistance of plants (Singh and Upadhyay, 2014). Evan’s Blue staining was performed on the transiently transformed birch plants after abiotic stress treatment. The results are shown in Figure 7C. Under the water treatment, there was no obvious color difference between OE plants, SE plants, the control group, and the WT, indicating that there was no remarkable difference in the degree of cell damage. However, under abiotic stress conditions, the damage of OE plant cell membranes was the lowest, and the damage of SE plant cell membranes was the highest. Our results indicate that *BpMYB4* can reduce the damage to cells in OE birch plants under abiotic stress. The Evan’s Blue staining results again showed that *BpMYB4* has a certain function of stress resistance in birch.

### BpMYB4 Regulates the Expression of Genes Related to Resistance and Cell Wall Biosynthesis

**FIGURE 8:**
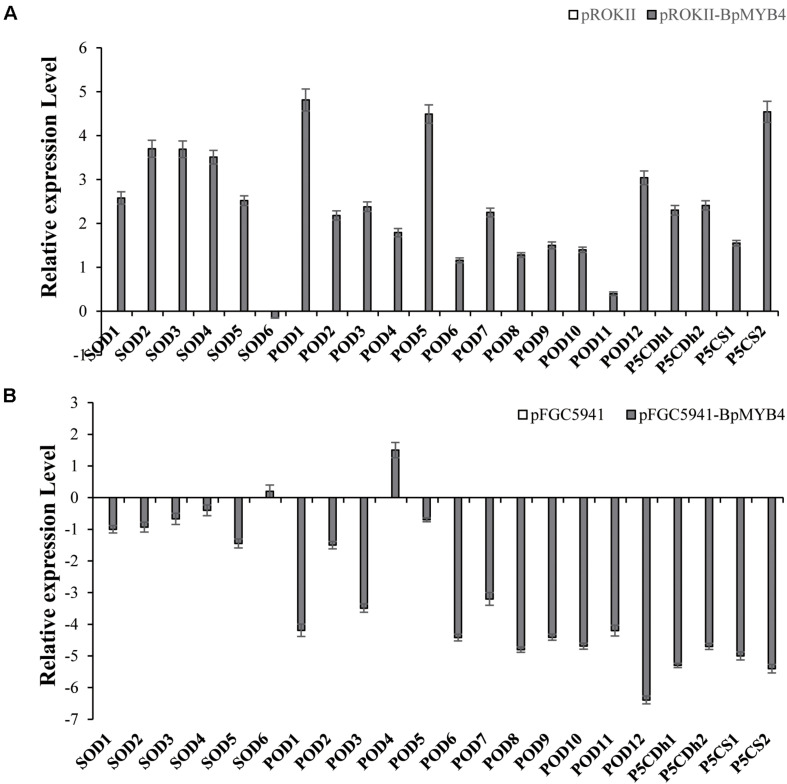
The expression levels of resistance-related genes in overexpression and suppression of expression in birch. **(A)** Taking the empty vector strain (pROKII) as a control, the expression levels of resistance-related genes in overexpressing strains were monitored. **(B)** Taking the empty vector strain (RNAi) as a control, the expression levels of resistance-related genes in the suppressive expression line were monitored.

**FIGURE 9:**
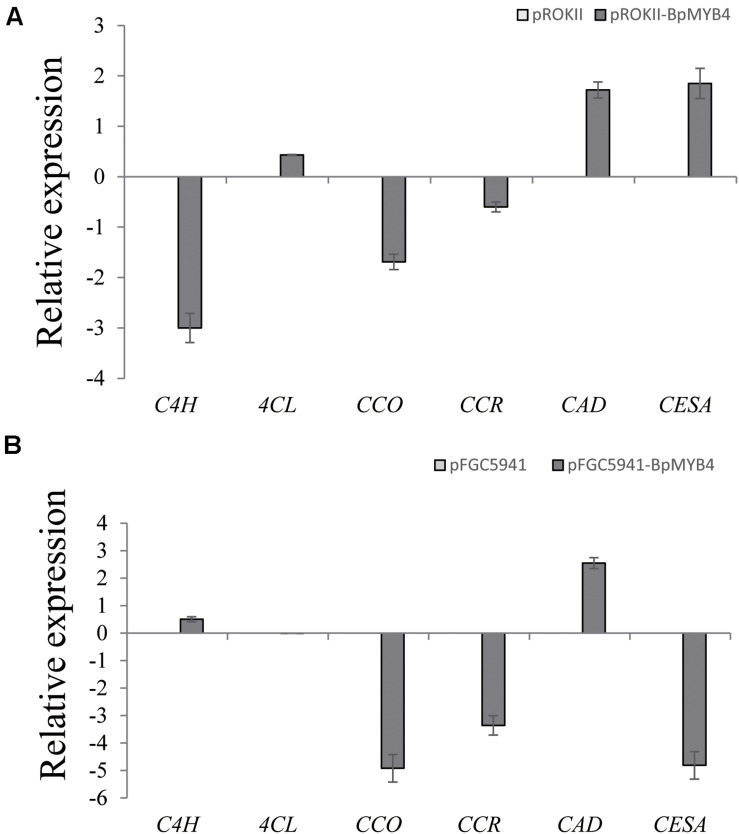
The expression levels ofcell wall biosynthesis-related genes in overexpression and suppression of expression in birch. **(A)** Taking the empty vector strain (pROKII) as a control, the expression levels of cell wall biosynthesis-related genes in overexpressing strains were monitored. **(B)** Taking the empty vector strain (RNAi) as a control, the expression levels of cell wall biosynthesis-related genes in the suppressive expression line were monitored.

To analyze the genes regulated by BpMYB4 in stress resistance and cell well formation, we analyzed the expression levels of putative downstream resistance-related and cell wall biosynthesis-related genes in overexpressing and knock-out transgenic lines. The results in Figure 8A show that most resistance-related genes were highly up-regulated in overexpressing lines (pROKII-*BpMYB4*) compared to the empty vector (pROK II) control line, including *SOD1, SOD2, SOD3, SOD4, SOD5, POD1, POD2, POD3 POD5, POD12, P5CDh1, P5CDh2*, and *P5CS2* genes. Among them, the maximum differential expression amount can reach as much as 27.4 fold, and the smallest is only about 1 flod. Therefore, most of the resistance-related genes were up-regulated in overexpressing lines, suggesting that overexpression of *BpMYB4* (pROKII-BpMYB4) can induce the expression of resistance-related genes. The results in Figure 8B show that most resistance genes are negatively regulated by the suppressed expression of *BpMYB4* (RNAi-BpMYB4), compared to the empty vector (RNAi) control line. Among them, *POD1, POD3, POD6, POD7, POD8, POD9, POD10, POD11, POD12, P5CDh1, P5CDh2, P5CS1*, and *P5CS2* expression were remarkably highly down-regulated. Among them, POD12 had the largest down-regulated expression level, and the down-regulated expression level could reach as much as 84.9 fold. These data indicate that knock-down expression of *BpMYB4* (RNAi-*BpMYB4*) can negatively affect the expression of resistance-related genes. The results in Figure 9A show that compared with the empty vector (pROKII) control line, in the OE lines (pROKII-*BpMYB4*), *CCO*, *CCR* and *C4H* genes which are the key regulator in lignin biosynthesis were down-regulated. *CESA* gene relate to cellulose biosynthesis was up-regulated, expression of *CAD* and *4CL* were also slight increased. Among them, the transcripts increase of *CESA* reached 3.61 fold, and the decreased expression level of *C4H* reached 8 fold. The results in Figure 9B show that compared with the RNAi empty vector (pFGC5941) control line, in the suppressed expression lines (pFGC5941-*BpMYB4*), *CAD* and *C4H* genes were up-regulated, and *CCO*, *CESA* and *CCR* genes were down-regulated. Among them, the increased expression level of *CAD* reached 5.86 fold, and the transcripts decrease of *CESA* reached 30.27 flod. These data suggest that BpMYB4 transcript factor can negatively affect the expression of some lignin biosynthesis gene and positive regulate the cellulose biosynthesis gene in OE plants compare with the WT. Therefore, *BpMYB4* has a certain function in regulating the expression of cell wall biosynthesis-related and resistance-related genes.

## Discussion

Multiple sequence alignment and phylogenetic tree analysis demonstrated that BpMYB4 is similar to EgMYB1, AmMYB308, ZmMYB42 and ZmMYB31 at the amino acid level, all of which have been identified as lignin biosynthetic inhibitory factors (Tamagnone et al., 1998; Fornalé et al., 2006, 2010; Legay et al., 2007). Therefore, we speculated that BpMYB4 might also play a negative role in lignin biosynthesis in birch.

According to relevant reports, genes encoding transcription factors that inhibit vascular development are usually not highly or specifically expressed in vascular tissues (Zhao and Dixon, 2011). In this study *BpMYB4* was expressed at high levels in flowers, at levels higher than that seen in stems. In addition, *BpMYB4* was abundantly expressed during the dormancy seasons of April and September. This expression pattern was unlike that seen in some genes whose products are involved in secondary cell wall formation, which were highly expressed in June or July (Wang et al., 2010a), and might imply the role of inhibitors in secondary growth. *BpMYB4* transcript levels increased during tension wood development compared with those seen in opposite wood and normal wood. In tension wood of birch, the cellulose content was higher and lignin content was lower than those in opposite wood and normal wood (Wang et al., 2014). Together with the expression level changes and phylogenetic tree analysis, our results suggest that *BpMYB4* might be an inhibitor of lignin biosynthesis in birch.

Studies in populations of forest tree hybrids have shown that when the lignin content is greatly reduced and the cellulose content is increased in transgenic plants, the growth rate of roots and stems is significantly increased (Hu et al., 1999). There is a negative correlation between biomass growth and lignin content (Kirst et al., 2004; Novaes et al., 2009, 2010). In the present study, the inflorescence stems of the transgenic plants was higher than the WT. So we detected the secondary xylem development and lignin deposition using anatomical observations (Figure 4) and chemical analysis. The stem sections stained with phloroglucinol-HCl showed decreased lignin deposition in transgenic plants, relative to WT *A. thaliana*. The content of lignin in transgenic lines was reduced, but the content of cellulose in transgenic plants was increased relative to WT (Table 1). Therefore, we hypothesized that the reason of growth increase in stems of transgenic plants is that *BpMYB4* inhibited lignin biosynthesis. These results confirmed that *BpMYB4* has a negative effect on lignin biosynthesis and deposition but a positive effect on cellulose content. The express analysis of cell wall biosynthesis relate genes in transgenetic birch with *BpMYB4* transiently over-expressing suggested that BpMYB4 transcript factor can decreased the expression of some lignin biosynthesis gene and positive regulate the cellulose biosynthesis gene in OE plants compare with the WT. These data might explain the mechanism of cell wall components modification in transgenetic plants.

The transgenetic birch with *BpMYB4* transiently expression were used to investigate the resistance ability of *BpMYB4*. On the one hand, functional analysis in birch was used to verify the results of heterologous expression of *BpMYB4* in Arabidopsis experiment, on the other hand, it is convenient to analyze the genes that can be regulated in birch by BpMYB4. ROS are produced when plants face adverse conditions (Scandalios, 1993). Levels of H_2_O_2_ in plant cells may accumulate and cause oxidative damage when plants undergo stress (Larrigaudière et al., 2001). The amount of H_2_O_2_ released from the cells can be detected by the intensity of DAB staining. Previous studies have shown that reduced H_2_O_2_ accumulation (detected via DAB) following abiotic stress can increase stress resistance (Sela et al., 2013). In this study, after treatment with 100 mM NaCl or 200 mM mannitol, DAB staining showed that H_2_O_2_ accumulation in OE plants was lower than WT, but higher in SE plants compared to WT. This indicates that BpMYB4 can reduce H_2_O_2_ accumulation in OE birch seedlings subjected to abiotic stress. Another way ROS is produced in cells is via stress signaling, the cellular level of O_2_^.–^ (Mittler et al., 2004). Staining with NBT indicates the activity of superoxide dismutase (Buc-Calderon and Roberfroid, 1988), which reflects the content of O_2_^.–^ in cells (Yu et al., 2018). In the present study, under abiotic stress conditions, the levels of O_2_^.–^ in OE plants were the lowest, and the levels of O_2_^.–^ in SE plants were the highest. The results indicate that BpMYB4 can reduce the accumulation of O_2_^.–^ in birch seedlings under abiotic stress. Accumulation of ROS may damage cell membranes by oxidation of proteins, lipids and DNA (Mittler et al., 2004). Studies have shown that Evan’s Blue staining can detect cell death and demonstrate the degree of stress resistance of plants (Singh and Upadhyay, 2014). Therefore, we performed Evan’s Blue staining on the transiently transformed birch plants after abiotic stress treatment. The results indicate that BpMYB4 can reduce the damage done by abiotic stress in OE lines of birch. BpMYB4 also induces the expression of most resistance-related genes. All the above results showed that BpMYB4 activates the metabolic pathway of ROS clearance by regulating the expression of resistance-related genes in transgenic birch trees subjected to abiotic stress, thereby reducing the level of ROS to maintain cell membrane integrity and playing a role in the resistance of birch trees.

## Conclusion

In this study, we identified a *BpMYB4* gene which is homologous to other transcription factors that negatively regulate lignin biosynthesis. The expression analysis of *BpMYB4* in different tissues, under artificial bending treatment and at different stages in a growing season also imply that it might be an inhibitory transcription factor in secondary growth. Functional analysis in transgenic Arabidopsis further demonstrated that *BpMYB4* can promote height growth of inflorescence stems, increase cellulose and decrease lignin content in the transgenic plants. Analysis of the stable transformation of Arabidopsis and transiently transformed birch with BpMYB4 also indicated a certain stress resistance function. These results can be useful for further understanding the molecular mechanism of secondary growth procesess and abiotic stress responses regulated by MYB transcription factors.

## Data Availability Statement

The datasets presented in this study can be found in online repositories. The names of the repository/repositories and accession number(s) can be found in the article/Supplementary Material.

## Author Contributions

CW: conceptualization and methodology. HL and NZ: software. YY, HL, and NZ: formal analysis and investigation. YY and CW: writing—review and editing. YY: visualization. CW: funding acquisition. All authors contributed to the article and approved the submitted version.

## Conflict of Interest

The authors declare that the research was conducted in the absence of any commercial or financial relationships that could be construed as a potential conflict of interest.

## Footnotes

1 https://blast.ncbi.nlm.nih.gov/Blast.cgi?PROGRAM~=~blastx&PAGE_TYPE~=~BlastSearch&LINK_LOC~=~blasthome

2 http://www.expasy.org/tools

## Supplementary Material

Supplementary Figure 1 Semi-quantitative PCR analysis of *BpMYB4* Gene in transgenic Arabidopsis.

Supplementary Figure 2 Observation of transgenic Arabidopsis phenotype. Comparison of transgenic Arabidopsis and WT at 1 week **(A)**, 4 weeks **(B)**, and 6 weeks **(C)**.

Supplementary Table 1 Vector construction primer sequence.

Supplementary Table 2 qRT-PCR primer sequence.
