# Supplementary figures and images for: The BpMYB4 Transcription Factor From Betula platyphylla Contributes Toward Abiotic Stress Resistance and Secondary Cell Wall Biosynthesis
Source: Front Plant Sci. 2021 Jan 18;11:606062. doi: 10.3389/fpls.2020.606062 (PMC7847980; doi:10.3389/fpls.2020.606062)

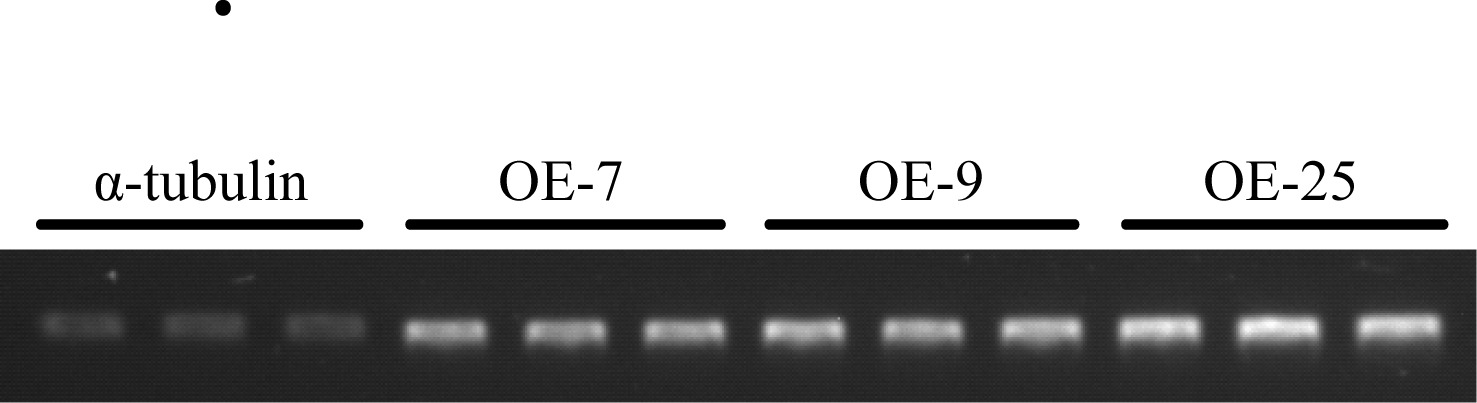

Supplement: Supplementary Figure 1 — Semi-quantitative PCR analysis of BpMYB4 Gene in transgenic Arabidopsis. [file Image_1.TIF]

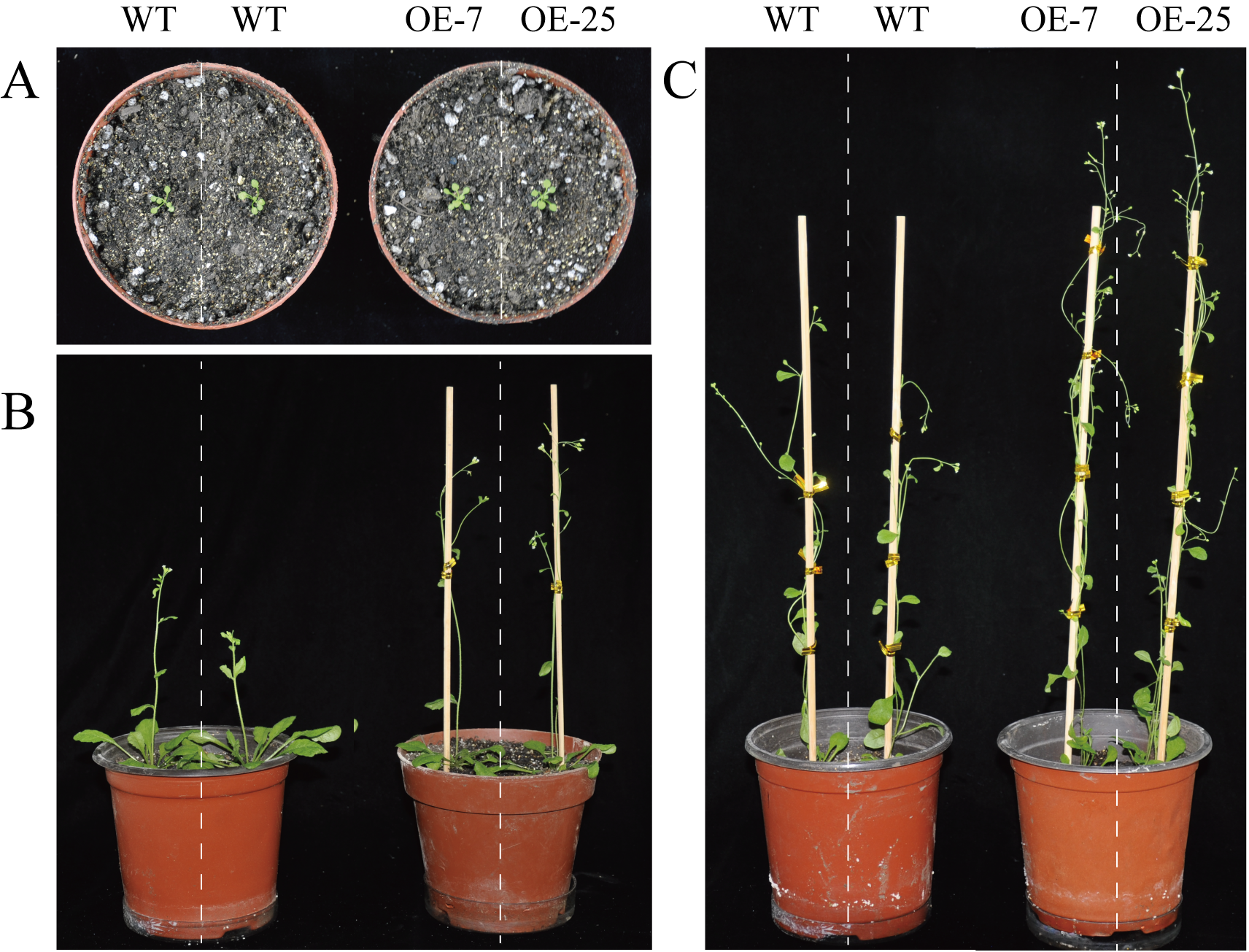

Supplement: Supplementary Figure 2 — Observation of transgenic Arabidopsis phenotype. Comparison of transgenic Arabidopsis and WT at 1 week (A), 4 weeks (B), and 6 weeks (C). [file Image_2.TIF]
