# Supplementary material for: The BpMYB4 Transcription Factor From Betula platyphylla Contributes Toward Abiotic Stress Resistance and Secondary Cell Wall Biosynthesis
Source: Front Plant Sci. 2021 Jan 18;11:606062. doi: 10.3389/fpls.2020.606062 (PMC7847980; doi:10.3389/fpls.2020.606062)
Supplement: Supplementary Table 1 — Vector construction primer sequence. [file Table_1.DOCX]

**Table S1.** Vector construction primer sequence

| **Primer** | **Sequence (5’-3’)** |
| --- | --- |
| MYB4-pROKⅡ-F | CGCGGATCCATGGGAAGGTCTCCTTGCTG |
| MYB4-pROKⅡ-R | CGGGGTACCTTACACATTAATCCTGCAGC |
| RNAi4-MYB4-F | CTAGTCTAGAGCAACCACTATATCTTTTGGTGC |
| RNAi-MYB4-R | CGCGGATCCATTCTGCAATCCCAAGCTGC |
| MYB4-GFP-F | CTCTAGAGGATCCCCATGGGAAGGTCTCCTTGCTGT |
| MYB4-GFP-R | TCGAGCTCGGTACCCCACATTAATCCTGCAGCTACAATC |
| pROKⅡ- F | GGCGAACGTGGCGAGAAAGG |
| pROKⅡ- R | ACAGGTTTCCCGACTGGAAAGC |
| RNAi-F | CGAGCTCGCAACCACTATATCTTTTGGTGC |
| RNAi-R | CTAGACTAGTATTCTGCAATCCCAAGCTGC |
