# Supplementary material for: The BpMYB4 Transcription Factor From Betula platyphylla Contributes Toward Abiotic Stress Resistance and Secondary Cell Wall Biosynthesis
Source: Front Plant Sci. 2021 Jan 18;11:606062. doi: 10.3389/fpls.2020.606062 (PMC7847980; doi:10.3389/fpls.2020.606062)
Supplement: Supplementary Table 2 — qRT-PCR primer sequence. [file Table_2.DOCX]

**Table S2.** qRT-PCR primer sequence

| **Primer** | **Sequence (5’-3’)** |
| --- | --- |
| BpMYB4-F | GGGGGAAGGACTCATTTTGT |
| BpMYB4-R | CCCCACCTGTTAAGTTCCAG |
| SOD1-F | CCGTGGTTGTTCTTGGCAAC |
| SOD1-R | CCAGCAGGATTGAAATGTGGC |
| SOD2-F | TGCTCCTAAAGCGGTGGTTC |
| SOD2-R | GGGTTGAAATGAGGGCCAGT |
| SOD3-F | GAAGCCGCACTAGTAGCCAT |
| SOD3-R | ACGACAGTGAGTGGTTTGGC |
| SOD4-F | CGGAGGTCATATCAACCACTC |
| SOD4-R | CAGACCAAGCCACACCCAT |
| SOD5-F | TCTGGAAGAATCTCGCACCC |
| SOD5-R | ATCAAGAGCAAGCCAAACCC |
| SOD6-F | CTTTGCTCTTTCCTCACTCT |
| SOD6-R | ACAACCCTCGCATTTCAT |
| POD1-F | ACTAAATCTACCACCACCC |
| POD1-R | TGTATCGCCCTTTCCAG |
| POD2-F | AGCATAAAAGAGCATCCCTC |
| POD2-R | CACATCCAGACCCAGAAATT |
| POD3-F | TTCATTGCGAGGGTTTG |
| POD3-R | TGCTTGAGAAGGCAGATAG |
| POD4-F | TACTGGACGCAAAGATGG |
| POD4-R | AGGAAGGAAAGCGGAAT |
| POD5-F | TGAGGTGAATGGGAAGAATG |
| POD5-R | AGGTGATGTGGTTGGTGC |
| POD6-F | CAAGTCGGCAGTTGAGAAT |
| POD6-R | TGGAAAGGCCCACATTAC |
| POD7-F | ACATCTTAGCCGTTGTTGC |
| POD7-R | GGCAGTGAGACCTTTGTTT |
| POD8-F | TGTCAACAATTCTGGGCTAC |
| POD8-R | CCACCCTACAGTTCTTCCT |
| POD9-F | AGGAACGAGAACGCTACTGC |
| POD9-R | AACACGATCCGATCTGATGC |
| POD10-F | GATGGCAGGGTATCTTTG |
| POD10-R | GCTTGGTTTATGGTGGG |
| POD11-F | GGCGTAGTCTCCTGCTCTG |
| POD11-R | GCTGCCCAAACATGGTAATG |
| POD12-F | GCTGTCAAGAACGCAGTGTC |
| POD12-R | ATGTACGGAGCGATTAGGTG |
| P5CS1-F | CAAACGCCACCTCACAGAC |
| P5CS1-R | TCGTGTAACAACGGCAGTCC |
| P5CS2-F | TGGGAACGGTCTACTGCTT |
| P5CS2-R | TTACTGCCTCTTGGGATTAC |
| P5CDH1-F | GACTTCAGCCTCCAACACCT |
| P5CDH1-R | TCAACCAGCCTAACCAACC |
| P5CDH2-F | TTCTATACCCTTTGCTACCG |
| P5CDH2-R | GAATGCCTGTTTCATCTACC |
| Ubiquitin F | GGAGGACAAGGTGGAGGG |
| Ubiquitin R | GATTGAGGGGAGGGATGC |
| α-tubulin F | TGGCTCGAATGCACTGTTGG |
| α-tubulin R | TCAACCGCCTTGTCTCTCAGG |
